# Supplementary material for: Ct threshold values, a proxy for viral load in community SARS-CoV-2 cases, demonstrate wide variation across populations and over time
Source: eLife. 2021 Jul 12;10:e64683. doi: 10.7554/eLife.64683 (PMC8282332; doi:10.7554/eLife.64683)
Supplement: Supplementary file 4. [file elife-64683-supp4.docx]

* SWAB_POS_ELIFE.DO

* analysis for paper on Positives and Ct Values

* - include all positives

* - add false-positives out of all tests at end

* updated for ELife revision March and April 2021

adopath + "P:\Working\ado/"

adopath + "P:\Working\user_asw\myado/"

* date of files and for analysis

global DATE "20210315"

global TODAY "15mar2021"

global WindowDate =d(13mar2021)

noi di _n "Swabs back from " %d $WindowDate

* UseDate is the Sunday AFTER WindowDate

global UseDate=d(14mar2021)

assert dow($UseDate)==0

assert inrange($UseDate-$WindowDate,0,6)

* directories

global MainData "P:/Working/user_statistical_processing/raw$DATE/Data"

global Data "P:/Working/user_asw/analysis/$DATE"

global Logs "P:/Working/user_asw/analysis/docs/2020 positives/analysis/eLife$DATE/Logs"

global Graphs "P:/Working/user_asw/analysis/docs/2020 positives/analysis/eLife$DATE/Graphs"

cap log close

log using "$Logs/swab_pos_paper_$DATE.log", replace

noi di "Run by ASW on $S_DATE $S_TIME"

noi version

noi di "Dataset: $DATE"

noi di "WindowDate: " %d $WindowDate

noi di "UseDate: " %d $UseDate

set more off

*************************************************************************************

* A: positives out of all tests

*************************************************************************************

noi di _n _dup(80) "*" _n _dup(80) "*" _n "A: All linked test results" _n _dup(80) "*" _n _dup(80) "*"

noi use participant_id result_mk visit_date visit_id lab_id country ageg_sy hh_id result_* assay_* n_participants_i swab_barcode_cleaned if result_mk<=1|result_tdi<=1 using $MainData/data_participant_clean, clear

assert visit_date<=$UseDate

* drop one duplicate positive from same person on same day - not in main dataset

sort participant visit_date result_mk swab_barcode_cleaned

noi by participant visit_date: drop if _n>1 & result_mk==1 & result_mk[1]==1 & inlist(participant,"DHR-200928237909")

by participant visit_date: assert _N==1|(result_mk[1]==0 & result_mk[2]==1 & _N==2 & inlist(participant,"DHR-200925221500")) if result_mk==1

* save as temporary file, as used below

tempfile main

save "`main'", replace

noi di _n "ALL POS/NEG SWABS to " %d $UseDate

* drop void results

drop if result_mk>=9

noi tab result_mk

qui summ result_mk if result_mk<=1

noi cii prop r(N) r(sum), exact

noi tab country result_mk

* first or subsequent test in study

preserve

gsort participant visit_date -result_mk

by participant: gen byte first=1 if _n==1

noi tab result_mk first if result_mk==1, miss

noi di _n "All participants"

noi tab result_mk first if first==1, col nokey

gen byte flag=(result_mk==1)

egen byte max=max(flag), by(participant)

egen int count=group(hh_id)

qui summ count

noi di _n "Number of households: " r(max)

drop if max==0

noi di _n "Participants with any positive"

noi tab result_mk first, col nokey

noi tab country first, col nokey

drop count

egen int count=group(hh_id)

qui summ count

noi di _n "Number of households with a positive: " r(max)

restore

* distribution of first test result, swabs per participant

sort participant visit_date

by participant: gen long first_test_month=mdy(month(visit_date),1,year(visit_date)) if _n==1

format first_test_month %d

noi tab first_test_month

by participant: gen byte n_swabs=_N if _n==1

noi tabstat n_swabs, by(first_test_month) c(s) s(median p25 p75 min max)

noi tab n_swabs

* number of prior negative tests

use $Data\swabpositives_evidence, clear

format ct_mean %7.1f

assert lab_id==2 if visit_date<d(16aug2020)

assert lab_id==1 if visit_date>d(9feb2021)

confirm var imd_pc_new imdg ageg_sy

* household classification - anyone else in the household positive EVER

bysort participant: gen byte flag=(_n==1)

egen byte hh_pos=sum(flag), by(hh_id)

drop flag

noi tab hh_pos

noi tab hh_pos n_participants

gen byte hh_pos_gt1=(hh_pos>1)

noi di _n "Number of prior negatives for first positive in the study"

noi tabstat count_prior_neg if any_prior_pos==0, by(first_test) c(s) s(n median p25 p75 min max)

noi datacheck count_prior_neg>0 if any_prior_pos==0 & first_test~=1, mess(Check numbers tie up with first test above) vars(participant first_test count_prior_neg count_prior_pos) noobs nod

*************************************************************************************

* B: Ct patterns and values overall

*************************************************************************************

noi di _n _dup(80) "*" _n _dup(80) "*" _n "B: Ct patterns, positive targets and values overall: Table 1" _n _dup(80) "*" _n _dup(80) "*"

noi tab count_pos_target, miss

noi di _n "Drop if missing Ct (non-standard lab)"

noi tab lab_id ct_mean if ct_mean>=., miss

noi drop if ct_mean>=.

noi tab count_pos_target, miss

noi di _n "Split count_pos_target by before or after 16nov (PHE technical briefing 2)"

noi replace count_pos_target=4 if count_pos_target==2 & ctpattern==4 & visit_date>=d(16nov2020)

label define count_pos_target 4 "OR/N 16nov-"

label values count_pos_target count_pos_target

noi tab count_pos_target

noi tab ctpattern count_pos_target

noi tab count_pos_target any_prior_pos, col nokey chi

gen byte CTPATTERN=ctpattern

replace CTPATTERN=8 if ctpattern==4 & visit_date>=d(16nov2020)

label define ctpattern 8 "OR+N 16nov-", add

label values CTPATTERN ctpattern

noi tab CTPATTERN any_prior_pos, col nokey chi

noi di _n "S only"

gen long visit_month=mdy(month(visit_date),1,year(visit_date))

format visit_month %d

noi tab visit_month if ctpattern==3

sort visit_date

noi datacheck ctpattern~=3 if visit_date>d(17may2020), mess(S only after 17 May 2020) vars(participant visit_date ctpattern ct_mean lab) noobs nod

noi di _n "Correlation between Ct values when called positive for both genes"

noi spearman ctNgene ctORF1ab if inlist(ctpattern,7,4)

noi spearman ctSgene ctORF1ab if inlist(ctpattern,7,5)

noi spearman ctNgene ctSgene if inlist(ctpattern,7,6)

noi di _n "Ct per sample: all positives"

noi tabstat ct_mean, by(count_pos_target) c(s) s(n median p25 p75 min max) format(%6.1f)

noi kwallis ct_mean, by(count_pos_target)

noi tabstat ct_mean, by(CTPATTERN) c(s) s(n median p25 p75 min max) format(%6.1f)

qreg ct_mean i.count_pos_target i.CTPATTERN

noi qreg

noi testparm i.CTPATTERN

noi di _n "Ct per sample: first positives"

noi tabstat ct_mean if any_prior_pos==0, by(count_pos_target) c(s) s(n median p25 p75 min max) format(%6.1f)

noi tabstat ct_mean if any_prior_pos==0, by(CTPATTERN) c(s) s(n median p25 p75 min max) format(%6.1f)

noi di _n "Ct over 37"

noi tab ct_mean if ct_mean>37

noi tab visit_month if ct_mean>37

noi tab visit_month lab if ct_mean>37

noi tab CTPATTERN lab if ct_mean>37

*************************************************************************************

* D: Ct & symptoms over calendar time

*************************************************************************************

noi di _n _dup(80) "*" _n _dup(80) "*" _n "D: Ct by symptoms and over time" _n _dup(80) "*" _n _dup(80) "*"

* use symptoms around test as this is what is most predictive

gen byte evidence_sympt=any_evidence_sympt_now

replace evidence_sympt=any_evidence_sympt_around

sort visit_date

noi datacheck evidence_sympt<., mess(Missing all symptoms - set to none for plots as all old) vars(participant visit_date count_pos ct_mean *evidence*) noobs nod

noi replace evidence_sympt=0 if evidence_sympt>=.

********

* violin plot of Ct and symptoms: Figure 1

********

gen byte temp=2*CTPATTERN+evidence_sympt-1

label define temp 1 "N only: no sympt" 2 "N only: sympt" 3 "OR only: no sympt" 4 "OR only: sympt" 5 "S only: no sympt" 6 "S only: sympt" /*

*/ 7 "OR+N -16nov: no sympt" 8 "OR+N -16nov: sympt" 9 "OR+S: no sympt" 10 "OR+S: sympt" 11 "N+S: no sympt" 12 "N+S: sympt" 13 "OR+N+S: no sympt" /*

*/ 14 "OR+N+S: sympt" 15 "OR+N 16nov-: no sympt" 16 "OR+N 16nov-: sympt"

label values temp temp

vioplot ct_mean, over(temp) graphregion(color(white)) ylabel(10(5)30 34 37, angle(0) format(%2.0f) labsize(small)) ytitle(Ct value) /*

*/ xtitle(" ") xlabel(,labsize(small) valuelabel angle(45)) xline(2.5(2)14.5,lpattern(dash) lcolor(gray) lw(*0.5))

graph export "$Graphs/violin_plot_fig_1.emf", replace

preserve

table CTPATTERN evidence_sympt, c(median ct_mean p25 ct_mean p75 ct_mean min ct_mean max ct_mean) replace

for num 1/5 \ any median p25 p75 min max: rename tableX Y

export excel using "$Logs/Figure1.xlsx", replace firstrow(var)

restore

********

* symptoms by Ct: Figure 2

********

preserve

replace ct_mean=round(ct_mean,1)

recode ct_mean 36/39=36 9/11=11

assert inrange(ct_mean,11,36)

compress

collapse (count) n=result_mk n_now=any_evidence_sympt_now n_around=any_evidence_sympt_around (sum) *cgh* any_evidence_sympt_now any_evidence_sympt_around, by(ct_mean)

local max=_N

foreach var of varlist any_evidence_sympt_now sympt_now_cgh {

gen `var'_p=100*`var'/n_now

for any l u: gen byte `var'_X=.

forval i=1(1)`max' {

local N=n_now[`i']

local n=`var'[`i']

qui cii prop `N' `n', exact

qui replace `var'_l=r(lb)*100 if _n==`i'

qui replace `var'_u=r(ub)*100 if _n==`i'

}

}

foreach var of varlist any_evidence_sympt_around sympt_around_cgh {

gen `var'_p=100*`var'/n_around

for any l u: gen byte `var'_X=.

forval i=1(1)`max' {

local N=n_around[`i']

local n=`var'[`i']

qui cii prop `N' `n', exact

qui replace `var'_l=r(lb)*100 if _n==`i'

qui replace `var'_u=r(ub)*100 if _n==`i'

}

}

rename any_evidence_sympt_now* now*

rename any_evidence_sympt_around* aro*

rename sympt_now_cghfevamn* now_cgh*

rename sympt_around_cghfevamn* aro_cgh*

gen plot1=ct_mean-0.15

gen plot2=ct_mean-0.05

gen plot3=ct_mean+0.05

gen plot4=ct_mean+0.15

twoway rcapsym now_u now_l plot2, color(black) lcolor(black) s(i) lwidth(thin) || /*

*/ scatter now_p plot2, color(black) lcolor(black) s(o) c(l) || /*

*/ rcapsym aro_u aro_l ct_mean, color(gs8) lcolor(gs8) s(i) lwidth(thin) || /*

*/ scatter aro_p ct_mean, color(gs8) lcolor(gs8) s(s) c(l) || /*

*/ rcapsym now_cgh_u now_cgh_l ct_mean, color(black) lcolor(black) s(i) lwidth(thin) || /*

*/ scatter now_cgh_p ct_mean, color(black) lcolor(black) s(o) c(l) mfcolor(white) || /*

*/ rcapsym aro_cgh_u aro_cgh_l plot3, color(gs8) lcolor(gs8) s(i) lwidth(thin) || /*

*/ scatter aro_cgh_p plot3, color(gs8) lcolor(gs8) s(s) c(l) mfcolor(white) || /*

*/ , graphregion(color(white)) ytitle(Percentage of positive tests (95% CI)) ylabel(0(10)100, angle(0)) /*

*/ xlabel(11(1)36, valuelabels labsize(vsmall) angle(45)) xtitle("Ct value (rounded)") /*

*/ legend(order(2 "Any evidence of symptoms at test" 6 "Cough, fever, anosmia at test" 4 "Any evidence of symptoms around test" 8 "Cough, fever, anosmia around test" ) size(vsmall) rows(2))

graph export "$Graphs\symptom_over_ct_mean_fig_2.emf", replace

export excel ct_mean now_p now_l now_u aro_p aro_l aro_u now_cgh_p now_cgh_l now_cgh_u aro_cgh_p aro_cgh_l aro_cgh_u using "$Logs\Figure2.xlsx", replace firstrow(var)

restore

*********

* Evidence: Table 2

*********

noi di _n "Level of evidence"

noi tab evidence

noi tab strength

noi tab strength if evidence==1

noi tab evidence count_pos_target, row nokey

noi tabstat ct_mean, by(evidence) c(s) s(n median p75 p90 p95 p99) format(%6.1f)

_pctile ct_mean if evidence==1, percentiles(90(1)97 97.5 98 99)

noi return list

noi tab evidence any_ct_under_34, row nokey

sort ct_mean

noi di _n "Factors determining evidence classification - symptoms and patient-facing/care work"

noi tab evidence any_evidence_sympt_around, row nokey chi

noi tab evidence any_evidence_sympt_around if evidence<=2, row nokey chi

gen byte occupation=max(ever_patient,ever_care_home)

noi tab evidence occupation, row nokey

noi tab evidence occupation if evidence<=2, row nokey chi

noi di _n "Other factors"

noi tab evidence sympt_now_cgh, row nokey chi

noi tab evidence sympt_around_cgh, row nokey chi

noi tab evidence sympt_around_cgh if evidence<=2, row nokey chi

noi tab evidence any_prior_pos, row nokey chi

noi tab evidence first_test, row nokey chi

noi tab evidence wgs_status if wgs_status<=2, row nokey chi

noi di _n "Ct in those positive one target with WGS"

noi tabstat ct_mean if streng==3, c(s) s(n median p75 p90 p95 p99) format(%6.1f)

noi tab evidence hh_pos_gt1, row nokey chi

noi di _n "- where >1 participant"

noi tab evidence hh_pos_gt1 if n_participants>1, row nokey chi

*************************************************************************************

* C: Predictors of Ct values

*************************************************************************************

noi di _n _dup(80) "*" _n _dup(80) "*" _n "C: Predictors of Ct values" _n _dup(80) "*" _n _dup(80) "*"

* use quantile regression

noi summ ct_mean, detail

* spline for date

noi spearman ct_mean visit_date

summ visit_date, detail

local min=r(min)

local max=r(max)

local p50=r(p50)

local p10=r(p10)-`p50'

local p90=r(p90)-`p50'

local p25=r(p25)-`p50'

local p75=r(p75)-`p50'

noi di _n "EQUALLY SPACED KNOTS for visit_date"

local p50=(`max'+`min')*0.5

local p10=`min'+(`max'-`min')*0.10-`p50'

local p90=`min'+(`max'-`min')*0.90-`p50'

local p25=`min'+(`max'-`min')*0.25-`p50'

local p75=`min'+(`max'-`min')*0.75-`p50'

gen long DATE=visit_date-`p50'

noi mkspline DATE_=DATE, cubic nknots(5) knots(`p10' `p25' 0 `p75' `p90') displayknots

qui qreg ct_mean DATE_*

noi qreg

noi testparm DATE_*

noi testparm DATE_2 DATE_3 DATE_4

* reset to zero on reference date

for num 2/4: summ DATE_X if DATE_1==0 \ replace DATE_X=DATE_X-r(mean)

noi di _n "Reference date " %d `p50'

* age

summ age_at_visit, detail

* truncate at 80

replace age_at=80 if age_at>80 & age_at<.

local min=r(min)

local max=r(max)

local p50=r(p50)

local p50=(`max'+`min')*0.5

local p10=`min'+(`max'-`min')*0.10-`p50'

local p90=`min'+(`max'-`min')*0.90-`p50'

local p25=`min'+(`max'-`min')*0.25-`p50'

local p75=`min'+(`max'-`min')*0.75-`p50'

gen long AGE=age_at_visit-`p50'

noi mkspline AGE_=AGE, cubic nknots(5) knots(`p10' `p25' 0 `p75' `p90') displayknots

drop AGE

* imd

summ imd_pc_new, detail

local min=r(min)

local max=r(max)

local p50=r(p50)

local p50=(`max'+`min')*0.5

local p10=`min'+(`max'-`min')*0.10-`p50'

local p90=`min'+(`max'-`min')*0.90-`p50'

local p25=`min'+(`max'-`min')*0.25-`p50'

local p75=`min'+(`max'-`min')*0.75-`p50'

gen long IMD=imd_pc_new-`p50'

noi mkspline IMD_=IMD, cubic nknots(5) knots(`p10' `p25' 0 `p75' `p90') displayknots

drop IMD

fvset base 3 ageg_sy

fvset base 5 imdg

foreach var of varlist count_pos first_test any_prior any_evidence* *split* sex ethnicityg ethnicity_wo ageg_sy imdg hh_pos_gt1 ever_patientfacing occupation ever_lthc smoke {

local mult=inlist("`var'","ageg_sy","ethnicityg","count_pos_target")

if strpos("`var'","split")>0 local mult=1

noi di _n _dup(80) "=" _n "`var' (UNIVARIABLE and BIVARIABLE WITH DATE)" _n _dup(80) "="

noi tabstat ct_mean, by(`var') c(s) s(n median p25 p75 min max) format(%6.1f)

cap assert `var'<.

if _rc~=0 {

noi di _n "In missings"

noi tabstat ct_mean if `var'>=., c(s) s(n median p25 p75 min max) format(%6.1f)

}

noi kwallis ct_mean, by(`var')

qui qreg ct_mean i.`var'

noi qreg

noi testparm i.`var'

if `mult'>0 noi testparm i.`var', equal

* adjusted for DATE

qui qreg ct_mean i.`var' DATE_*

noi qreg

noi testparm i.`var'

if `mult'>0 noi testparm i.`var', equal

if "`var'"=="ageg_sy" {

noi spearman ct_mean age_at_visit

qui qreg ct_mean age_at_visit

noi qreg

qui qreg ct_mean age_at_visit DATE_*

noi qreg

qui qreg ct_mean AGE_*

noi qreg

noi testparm AGE*

noi testparm AGE_2 AGE_3 AGE_4

qui qreg ct_mean AGE_* DATE_*

noi qreg

noi testparm AGE*

noi testparm AGE_2 AGE_3 AGE_4

}

else if "`var'"=="imdg" {

noi spearman ct_mean imd_pc_new

qui qreg ct_mean imd_pc_new

noi qreg

qui qreg ct_mean imd_pc_new DATE_*

noi qreg

qui qreg ct_mean IMD_*

noi qreg

noi testparm IMD_*

noi testparm IMD_2 IMD_3 IMD_4

qui qreg ct_mean IMD_* DATE_*

noi qreg

noi testparm IMD_*

noi testparm IMD_2 IMD_3 IMD_4

}

else if "`var'"=="hh_pos_gt1" {

noi spearman ct_mean hh_pos

qui qreg ct_mean hh_pos

noi qreg

}

}

**********************************************************************************************************************

noi di _n _dup(80) "*" _n _dup(80) "*" _n "FULL MULTIVARIABLE MODEL with SYMPTOMS AT TEST" _n _dup(80) "*" _n _dup(80) "*"

**********************************************************************************************************************

qui qreg ct_mean i.count_pos_target i.any_evidence_sympt_now i.any_prior_pos i.first_test i.sex i.ethnicity_wo

noi qreg

noi testparm i.count_pos_target

noi testparm 2.count_pos_target

noi testparm 3.count_pos_target

noi testparm 4.count_pos_target

noi testparm i.count_pos_target, equal

noi testparm 4.count_pos_target 3.count_pos_target, equal

noi margins i.count_pos_target, atmeans

foreach var of varlist any_evidence_sympt_now any_prior_pos first_test sex ethnicity_wo {

noi testparm i.`var'

noi margins i.`var'

}

noi di _n _dup(80) "=" _n "WITH DATE" _n _dup(80) "="

qui qreg ct_mean i.count_pos_target i.any_evidence_sympt_now i.any_prior_pos i.first_test i.sex i.ethnicity_wo DATE_*

noi qreg

noi testparm DATE_*

noi di _n _dup(80) "=" _n "SPLITTING SYMPTOMS" _n _dup(80) "="

qui qreg ct_mean i.count_pos_target i.sympt_now_split i.any_prior_pos i.first_test i.sex i.ethnicity_wo

noi qreg

noi testparm i.sympt_now_split

noi testparm i.sympt_now_split, equal

noi testparm 1.sympt_now_split

noi testparm 2.sympt_now_split

noi margins i.sympt_now_split

noi di _n _dup(80) "=" _n "Effect of other variables" _n _dup(80) "="

foreach var of varlist hh_pos_gt1 ethnicityg ageg_sy imdg ever_patientfacing occupation ever_lthc smoke {

qui qreg ct_mean i.count_pos_target i.any_evidence_sympt_now i.any_prior_pos i.first_test i.sex i.ethnicity_wo i.`var'

noi qreg

noi testparm i.`var'

*noi margins i.`var'

}

foreach thing in age_at_visit AGE_* imd_pc_new IMD_* {

qui qreg ct_mean i.count_pos_target i.any_evidence_sympt_now i.any_prior_pos i.first_test i.sex i.ethnicity_wo `thing'

noi qreg

noi testparm `thing'

}

noi di _n _dup(80) "=" _n "Mixed model with RE for hh_id - check similar" _n _dup(80) "="

qui mixed ct_mean i.count_pos_target i.any_evidence_sympt_now i.any_prior_pos i.first_test i.sex i.ethnicity_wo || hh_id: || participant_id:

noi mixed

qui mixed ct_mean i.count_pos_target i.any_evidence_sympt_now i.any_prior_pos i.first_test i.sex i.ethnicity_wo || hh_id:

noi mixed

**********************************************************************************************************************

noi di _n _dup(80) "=" _n _dup(80) "=" _n "FULL MULTIVARIABLE MODEL with SYMPTOMS AROUND TEST (more people)" _n _dup(80) "=" _n _dup(80) "="

**********************************************************************************************************************

qui qreg ct_mean i.count_pos_target i.any_evidence_sympt_around i.any_prior_pos i.first_test i.sex i.ethnicity_wo

noi qreg

noi testparm i.count_pos_target

noi testparm 2.count_pos_target

noi testparm 3.count_pos_target

noi testparm 4.count_pos_target

noi testparm i.count_pos_target, equal

noi testparm 4.count_pos_target 3.count_pos_target, equal

noi margins i.count_pos_target, atmeans

foreach var of varlist any_evidence_sympt_around any_prior_pos first_test sex ethnicity_wo {

noi testparm i.`var'

noi margins i.`var'

}

noi di _n _dup(80) "=" _n "WITH DATE" _n _dup(80) "="

qui qreg ct_mean i.count_pos_target i.any_evidence_sympt_around i.any_prior_pos i.first_test i.sex i.ethnicity_wo DATE_*

noi qreg

noi testparm DATE_*

noi di _n _dup(80) "=" _n "SPLITTING SYMPTOMS" _n _dup(80) "="

qui qreg ct_mean i.count_pos_target i.sympt_around_split i.any_prior_pos i.first_test i.sex i.ethnicity_wo

noi qreg

noi testparm i.sympt_around_split

noi testparm i.sympt_around_split, equal

noi testparm 1.sympt_around_split

noi testparm 2.sympt_around_split

noi margins i.sympt_around_split

noi di _n _dup(80) "=" _n "Effect of other variables" _n _dup(80) "="

foreach var of varlist hh_pos_gt1 ethnicityg ageg_sy imdg ever_patientfacing occupation ever_lthc smoke {

qui qreg ct_mean i.count_pos_target i.any_evidence_sympt_around i.any_prior_pos i.first_test i.sex i.ethnicity_wo i.`var'

noi qreg

noi testparm i.`var'

*noi margins i.`var'

}

foreach thing in age_at_visit AGE_* imd_pc_new IMD_* {

qui qreg ct_mean i.count_pos_target i.any_evidence_sympt_around i.any_prior_pos i.first_test i.sex i.ethnicity_wo `thing'

noi qreg

noi testparm `thing'

}

noi di _n _dup(80) "=" _n "Mixed model with RE for hh_id - check similar" _n _dup(80) "="

qui mixed ct_mean i.count_pos_target i.any_evidence_sympt_around i.any_prior_pos i.first_test i.sex i.ethnicity_wo || hh_id: || participant_id:

noi mixed

qui mixed ct_mean i.count_pos_target i.any_evidence_sympt_around i.any_prior_pos i.first_test i.sex i.ethnicity_wo || hh_id:

noi mixed

**********************************************************************************************************************

noi di _n _dup(80) "*" _n _dup(80) "*" _n "EXCL SYMPTOMS, COUNT: FULL MULTIVARIABLE MODEL with SYMPTOMS AT TEST" _n _dup(80) "*" _n _dup(80) "*"

**********************************************************************************************************************

qui qreg ct_mean i.any_prior_pos i.first_test i.sex i.ethnicity_wo i.ageg_sy i.imdg

noi qreg

foreach var of varlist any_prior_pos first_test sex ethnicity_wo ageg_sy imdg {

noi testparm i.`var'

noi margins i.`var'

}

qui qreg ct_mean i.any_prior_pos i.first_test i.sex i.ethnicity_wo i.ageg_sy i.imdg DATE_*

noi qreg

qui qreg ct_mean i.any_prior_pos i.first_test i.sex i.ethnicity_wo AGE_* imd_pc_new

noi qreg

foreach var of varlist any_prior_pos first_test sex ethnicity_wo {

noi testparm i.`var'

noi margins i.`var'

}

noi testparm imd_pc_new

noi lincom 20*imd_pc_

* marginal effect of age

summ any_prior_pos

local b_prior=r(mean)

summ first_test

local b_first=r(mean)

summ sex

local b_sex=r(mean)-1

summ ethnicity_wo

local b_eth=r(mean)-1

summ imd_pc

local b_imd=r(mean)

gen plot_age=_b[_cons]+`b_prior'*_b[1.any_prior_pos]+`b_first'*_b[1.first_test]+`b_sex'*_b[1.sex]+`b_eth'*_b[1.ethnicity_wo]+`b_imd'*_b[imd_pc_new ]

for num 1/4: replace plot_age=plot_age+AGE_X*_b[AGE_X]

noi di _n _dup(80) "=" "Effect of other variables" _n _dup(80) "="

foreach var of varlist hh_pos_gt1 ethnicityg ever_patientfacing occupation ever_lthc smoke {

qui qreg ct_mean i.any_prior_pos i.first_test i.sex i.ethnicity_wo AGE_* imd_pc_new i.`var'

noi qreg

noi testparm i.`var'

}

foreach thing in IMD_2-IMD_4 DATE_* {

qui qreg ct_mean i.any_prior_pos i.first_test i.sex i.ethnicity_wo AGE_* imd_pc_new `thing'

noi qreg

noi testparm `thing'

}

*************************************************************************************

* G: CT, symptoms, evidence over time

*************************************************************************************

noi di _n _dup(80) "=" _n "G: CT, symptoms, evidence over time" _n _dup(80) "="

noi tab wofy

preserve

collapse (p10) p10=ct_mean (p25) p25=ct_mean (p50) p50=ct_mean (p75) p75=ct_mean (p90) p90=ct_mean (mean) mean=ct_mean (min) visit_date (count) n=ct_mean, by(wofy WOFY)

summ wofy

local min=r(min)

local max=r(max)

gen byte y=40 if mod(wofy,2)==0

replace y=39 if mod(wofy,2)==1

format n %4.0f

scatter mean p10 p25 p50 p75 p90 wofy, c(l..) s(i..) lp(dash solid..) color(red navy blue orange purple gray) || /*

*/ scatter y wofy, s(i) c(i) mlabel(n) mlabcolor(black) mlabpos(0) mlabsize(*0.6) || /*

*/ , graphregion(color(white)) ylabel(10(5)35 37 40 "N", angle(0) format(%2.0f) labsize(small)) ytitle(Ct value) /*

*/ xtitle("Week starting (2020/1)") xlabel(`min'(2)`max', valuelabels labsize(vsmall) angle(45)) /*

*/ legend(order(1 "Mean" - "Percentiles" 2 "10th" 3 "25th" 4 "50th" 5 "75th" 6 "90th") rows(1) size(vsmall) keygap(*0.1) symxsize(*0.5))

graph export "$Graphs/ct_monitoring_fig_3A_UK.emf", replace

export excel wofy mean p10 p25 p50 p75 p90 using "$Logs/Figure3A.xlsx", replace firstrow(var)

restore

preserve

keep if country==0

collapse (p10) p10=ct_mean (p25) p25=ct_mean (p50) p50=ct_mean (p75) p75=ct_mean (p90) p90=ct_mean (mean) mean=ct_mean (min) visit_date (count) n=ct_mean, by(wofy WOFY)

gen byte y=40 if mod(wofy,2)==0

replace y=39 if mod(wofy,2)==1

format n %4.0f

summ wofy

local min=r(min)

local max=r(max)

* England lockdowns 4nov-1dec; 20dec-8 march; first one schools open 1 June

gen byte upper=38 if inrange(wofy,45,49)

gen byte upper2=38 if inrange(wofy,52,62)

gen byte upper3=38 if inrange(wofy,18,23)

gen byte upper4=38 if inrange(wofy,62,65)

gen byte lower=10 if inrange(wofy,45,49)

gen byte lower2=10 if inrange(wofy,52,62)

gen byte lower3=10 if inrange(wofy,18,23)

gen byte lower4=10 if inrange(wofy,62,65)

twoway rarea upper lower wofy, color(gs15) || /*

*/ rarea upper4 lower4 wofy, color(gs15) || /*

*/ rarea upper2 lower2 wofy, color(gs13) || /*

*/ rarea upper3 lower3 wofy, color(gs13) || /*

*/ scatter mean p10 p25 p50 p75 p90 wofy, c(l..) s(i..) lp(dash solid..) color(red navy blue orange purple gray) || /*

*/ scatter y wofy, s(i) c(i) mlabel(n) mlabcolor(black) mlabpos(0) mlabsize(*0.6)|| /*

*/ , graphregion(color(white)) ylabel(10(5)35 37 40 "N", angle(0) format(%2.0f) labsize(small)) ytitle(Ct value (England only)) /*

*/ xtitle("Week starting (2020/1) (England only)") xlabel(`min'(2)`max', valuelabels labsize(vsmall) angle(45)) /*

*/ legend(order(5 "Mean" - "Percentiles" 6 "10th" 7 "25th" 8 "50th" 9 "75th" 10 "90th") rows(1) size(vsmall) keygap(*0.1) symxsize(*0.5))

graph export "$Graphs/ct_monitoring_fig_3B_Eng.emf", replace

export excel wofy mean p10 p25 p50 p75 p90 using "$Logs/Figure3B_Eng.xlsx", replace firstrow(var)

restore

* symptoms & evidence by WEEK

gen byte high=(evidence==1)

gen byte mod=(evidence==2)

gen byte low=(evidence==3)

* NUMBERS TOO FEW INITIALLY - have to group

preserve

recode wofy 19=18 21=20 23=22 25=24 27=26 29=28 31=30 33=32 35=34

collapse (count) n=result_mk n_now=any_evidence_sympt_now n_around=any_evidence_sympt_around (sum) *evidence* *cgh* high mod low, by(wofy)

local max=_N

foreach var of varlist high mod low {

gen `var'_p=100*`var'/n

for any l u: gen byte `var'_X=.

forval i=1(1)`max' {

local N=n[`i']

local n=`var'[`i']

qui cii prop `N' `n', exact

qui replace `var'_l=r(lb)*100 if _n==`i'

qui replace `var'_u=r(ub)*100 if _n==`i'

}

}

foreach var of varlist any_evidence_sympt_now sympt_now_cgh {

gen `var'_p=100*`var'/n_now

for any l u: gen byte `var'_X=.

forval i=1(1)`max' {

local N=n_now[`i']

local n=`var'[`i']

qui cii prop `N' `n', exact

qui replace `var'_l=r(lb)*100 if _n==`i'

qui replace `var'_u=r(ub)*100 if _n==`i'

}

}

foreach var of varlist any_evidence_sympt_around sympt_around_cgh {

gen `var'_p=100*`var'/n_around

for any l u: gen byte `var'_X=.

forval i=1(1)`max' {

local N=n_around[`i']

local n=`var'[`i']

qui cii prop `N' `n', exact

qui replace `var'_l=r(lb)*100 if _n==`i'

qui replace `var'_u=r(ub)*100 if _n==`i'

}

}

rename any_evidence_sympt_now* now*

rename any_evidence_sympt_around* aro*

rename sympt_now_cghfevamn* now_cgh*

rename sympt_around_cghfevamn* aro_cgh*

gen byte y=80 if mod(wofy,2)==0

replace y=78 if mod(wofy,2)==1

gen int y2=105 if mod(wofy,2)==0

replace y2=103 if mod(wofy,2)==1

gen plot1=wofy-0.2

gen plot2=wofy-0.2/3

gen plot3=wofy+0.2/3

gen plot4=wofy+0.2

for var plot*: label values X wofy

summ wofy

local min=r(min)

local max=r(max)

twoway rcapsym now_u now_l plot1, color(black) lcolor(black) s(i) lwidth(thin) || /*

*/ scatter now_p plot1, color(black) lcolor(black) s(o) c(l) || /*

*/ rcapsym aro_u aro_l plot2, color(gs8) lcolor(gs8) s(i) lwidth(thin) || /*

*/ scatter aro_p plot2, color(gs8) lcolor(gs8) s(s) c(l) || /*

*/ rcapsym now_cgh_u now_cgh_l plot3, color(black) lcolor(black) s(i) lwidth(thin) || /*

*/ scatter now_cgh_p plot3, color(black) lcolor(black) s(o) c(l) mfcolor(white) || /*

*/ rcapsym aro_cgh_u aro_cgh_l plot4, color(gs8) lcolor(gs8) s(i) lwidth(thin) || /*

*/ scatter aro_cgh_p plot4, color(gs8) lcolor(gs8) s(s) c(l) mfcolor(white) || /*

*/ scatter y wofy, s(i) c(i) mlabel(n) mlabcolor(black) mlabpos(0) mlabsize(*0.6)|| /*

*/ , graphregion(color(white)) ytitle(Percentage of positive tests (95% CI)) ylabel(0(10)70 80 "N", angle(0) labsize(small)) /*

*/ xlabel(`min'(2)`max', valuelabels labsize(vsmall) angle(45)) xtitle("") /*

*/ legend(order(2 "Any evidence of symptoms at test" 6 "Cough, fever, anosmia at test" 4 "Any evidence of symptoms around test" 8 "Cough, fever, anosmia around test" ) size(vsmall) rows(2))

graph export "$Graphs\symptom_over_wofy_fig_3C.emf", replace

export excel wofy now_p now_l now_u aro_p aro_l aro_u now_cgh_p now_cgh_l now_cgh_u aro_cgh_p aro_cgh_l aro_cgh_u using "$Logs\Figure3C.xlsx", replace firstrow(var)

replace plot1=wofy-0.2

replace plot3=wofy+0.2

twoway rcapsym high_u high_l plot1, color(black) lcolor(black) s(i) lwidth(thin) || /*

*/ scatter high_p plot1, color(black) lcolor(black) s(s) c(l) || /*

*/ rcapsym mod_u mod_l wofy, color(gs8) lcolor(gs8) s(i) lwidth(thin) || /*

*/ scatter mod_p wofy, color(gs8) lcolor(gs8) s(d) c(l) mfcolor(white) || /*

*/ rcapsym low_u low_l plot3, color(gs13) lcolor(gs13) s(i) lwidth(thin) || /*

*/ scatter low_p plot3, color(gs13) lcolor(gs13) s(t) c(l) mfcolor(white) || /*

*/ scatter y2 wofy, s(i) c(i) mlabel(n) mlabcolor(black) mlabpos(0) mlabsize(*0.6) || /*

*/ , graphregion(color(white)) ytitle(Percentage of positive tests (95% CI)) ylabel(0(10)100 105 "N", angle(0) labsize(small)) /*

*/ xlabel(`min'(2)`max', valuelabels labsize(vsmall) angle(45)) xtitle("") /*

*/ legend(order(2 "Higher evidence" 4 "Moderate evidence" 6 "Lower evidence" ) size(vsmall) rows(1))

graph export "$Graphs\evidence_over_wofy_fig_3D.emf", replace

export excel wofy high_p high_l high_u mod_p mod_l mod_u low_p low_l low_u using "$Logs\Figure3D.xlsx", replace firstrow(var)

restore

* Ct distribution by evidence over time

* NUMBERS TOO FEW INITIALLY - have to group

preserve

recode wofy 19=18 21=20 23=22 25=24 27=26 29=28 31=30 33=32 35=34

collapse (p25) p25=ct_mean (p50) p50=ct_mean (p75) p75=ct_mean (count) n=ct_mean, by(wofy evidence)

reshape wide p25 p50 p75 n, i(wofy) j(evidence)

summ wofy

local min=r(min)

local max=r(max)

scatter p501 p502 p503 wofy, c(l..) s(i..) lp(dash solid..) color(black gs8 gs13) || /*

*/ , graphregion(color(white)) ylabel(10(5)35 37, angle(0) format(%2.0f) labsize(small)) ytitle(Median Ct value) /*

*/ xtitle("Week starting (2020/1)") xlabel(`min'(2)`max', valuelabels labsize(vsmall) angle(45)) /*

*/ legend(order(1 "Higher evidence" 2 "Moderate evidence" 3 "Lower evidence") rows(1) size(vsmall) keygap(*0.1) symxsize(*0.5))

graph export "$Graphs/ct_by_evidence_fig_4A.emf", replace

for num 1/3 \ any high mod low: rename p50X median_Y

export excel wofy median_high median_mod median_low using "$Logs\Figure4A.xlsx", replace firstrow(var)

restore

*************************************************************************************

* F: Antibody response

*************************************************************************************

noi di _n _dup(80) "=" _n _dup(80) "=" _n "F: Antibody response - first positive only" _n _dup(80) "=" _n _dup(80) "="

keep participant visit_date

sort participant visit_date

by participant: keep if _n==1

rename visit_date first_pos_date

merge 1:m participant using "`main'", update

drop if _m==2

assert _m==3

drop _m

* remove failed results

replace result_tdi=. if result_tdi>1

gen byte flag=(result_tdi<=1)

egen byte any_ab=max(flag), by(participant)

egen byte count_ab=sum(flag), by(participant)

drop flag

bysort participant (visit_date): gen byte first=1 if _n==1

noi tab any_ab if first==1

noi tab count_ab if first==1

noi tab count_ab if first==1 & count_ab>1

keep if result_tdi<.

* check no duplicates ab on same day

gsort participant visit_date -result_tdi

by participant visit_date: assert _N==1

* plots of antibody values

* one participant has non-usual lab positive test (no Ct) BEFORE this test

noi datacheck visit_date>=first_pos_date if result_mk==1, mess(Swab positive BEFORE first swab positive date - dropped positive from Loughborough) vars(participant visit_date result_mk first_pos_date) noobs nod

noi replace first_pos_date=d(16dec2020) if first_pos_date==d(22dec2020) & participant=="DHR-200913152768"

gen int day=visit_date-first_pos_date

noi summ day, det

* truncate at 5th and 95th percentiles closest to multiples of 30

local lower=-120

local upper=150

sort participant day

* keep at most one at the end and beginning to stop vertical lines

by participant: drop if _n<_N & day<`lower' & day[_n+1]<=`lower'

by participant: drop if _n>1 & day>`upper' & day[_n-1]>=`upper'

replace day=`lower' if day<`lower'

replace day=`upper' if day>`upper'

* count numbers before and after first pos

for any min max: egen int X=X(day), by(participant)

drop first

bysort participant (visit_date): gen byte first=1 if _n==1

* bin responses

gen int DAY=day

for num `lower'(30)`upper': replace DAY=X if inrange(day,X,X+29)

summ DAY

replace DAY=DAY-30 if DAY==r(max)

replace DAY=14 if inrange(day,14,29)

label define DAY -120 "[-120,-91]" -90 "[-90,-61]" -60 "[-60,-31]" -30 "[-30,-1]" 0 "[0,13]" 14 "[14,29]" 30 "[30,59]" 60 "[60,89]" 90 "[90,119]" 120 "[120,150]"

label values DAY DAY

label var DAY "Days from first positive swab"

noi di _n "All results"

noi tab DAY result_tdi, row nokey

* any positive on or after swab positive

gen byte flag=result_tdi*(visit_date>=first_pos_date)

egen byte any_pos_ab=max(flag), by(participant)

label var any_pos_ab "Positive Ab at or after 1st pos swab"

* any negative within 120 days before negative to 21 days post negative

replace flag=(result_tdi==0)*inrange(visit_date,first_pos_date-120,first_pos_date+21)

egen byte any_neg_ab=max(flag), by(participant)

label var any_neg_ab "Negative Ab in[-120,+21] of 1st pos swab"

drop flag

noi di _n "Latest positive per window"

sort participant DAY result_tdi day

noi by participant DAY: keep if _n==_N

noi tab DAY result_tdi, row nokey

drop first

bysort participant (visit_date): gen byte first=1 if _n==1

egen byte postonly=min(DAY), by(participant)

replace postonly=(postonly>=0)

label define postonly 0 "Antibody result(s) before 1st swab-positive (day 0)" 1 "No antibody result(s) before 1st swab-positive (day 0)"

label values postonly postonly

noi tab postonly first

noi tab postonly any_pos_ab if first==1

noi tab postonly any_neg_ab if first==1, cell nokey

noi bysort postonly: tab DAY result_tdi, row nokey

noi bysort DAY: tab postonly result_tdi, row nokey chi

gen byte i=1

graph export "$Graphs/swab_positive_ab_over_time_perc_fig_S4A.emf", replace

graph bar (count) i, subtitle(, size(small)) by(postonly, graphregion(color(white)) note("")) over(result_tdi) asyvars over(DAY, label(angle(45) labsize(small))) stack percentage bar(1, color(white) lcolor(black)) bar(2, color(black)) ytitle(Percentage of participants with antibody tests) ylabel(0(20)100, angle(0)) legend(order(1 "Negative" 2 "Positive") rows(1))

graph export "$Graphs/fig_5.emf", replace

collapse (sum) i, by(postonly DAY result_tdi)

reshape wide i, i(postonly DAY) j(result_tdi)

gen perc=100*i1/(i1+i0)

export excel postonly DAY perc using "$Logs\Figure5.xlsx", replace firstrow(var)

*************************************************************************************

* H: All tests

*************************************************************************************

noi di _n _dup(80) "=" _n "H: all tests by wofy" _n _dup(80) "="

use if result_mk<=1 using "`main'", clear

merge 1:1 participant visit_id using $Data\swabpositives_evidence, keepusing(evidence)

assert inlist(_m,1,3)

drop _m

assert inlist(result_mk,0,1)

assert dow(d(1jan2020))==3

gen int dofy=visit_date-d(1jan2020)+2 if year(visit_date)==2020

gen int wofy=1+int(dofy/7)

label values wofy wofy

* 2021

assert dow(d(1jan2021))==5

assert wofy==53 if visit_date==d(31dec2020)

replace wofy=53 if inrange(visit_date,d(1jan2021),d(3jan2021))

replace dofy=visit_date-d(4jan2021) if visit_date>d(3jan2021)

replace wofy=54+int(dofy/7) if visit_date>d(3jan2021)

* 26 April is only 1 person - reset

summ wofy

assert r(min)==17

summ result_mk if wofy==17 & result_mk==1

assert r(N)==1

recode wofy 17=18

gen int WOFY=wofy

assert WOFY<.

drop dofy

noi tab wofy

noi tab wofy result_mk

* evidence by wofy

gen byte high=(evidence==1)

gen byte mod=(evidence==2)

gen byte low=(evidence==3)

* NUMBERS TOO FEW INITIALLY - have to group even more

recode wofy 18/20=18 21/23=21 24/26=24 27/29=27 30/32=30 33/35=33

collapse (count) n=result_mk (sum) high mod low, by(wofy)

local max=_N

foreach var of varlist high mod low {

gen `var'_p=100*`var'/n

for any l u: gen byte `var'_X=.

forval i=1(1)`max' {

local N=n[`i']

local n=`var'[`i']

qui cii prop `N' `n', exact

qui replace `var'_l=r(lb)*100 if _n==`i'

qui replace `var'_u=r(ub)*100 if _n==`i'

}

}

for any high_p mod_p low_p: gen str6 Xstr=string(X,"%4.3f")+"%"

*\ replace Xstr=subinstr(Xstr,"0.",".",.)

gen plot1=wofy-0.3

gen plot2=wofy+0.3

for var plot*: label values X wofy

* zero low - plot at lowest other point

summ low_l if low_l>0

local min=r(min)

assert `min'>0.0005

for var low_l low_p: replace X=`min' if X<=0.0005

summ wofy

local min=r(min)

local max=r(max)

* line plot rather than bar chart

twoway rcapsym high_u high_l plot1, color(black) lcolor(black) s(i) lwidth(thin) || /*

*/ scatter high_p plot1, color(black) lcolor(black) s(s) c(l) || /*

*/ rcapsym mod_u mod_l wofy, color(gs8) lcolor(gs8) s(i) lwidth(thin) || /*

*/ scatter mod_p wofy, color(gs8) lcolor(gs8) s(d) c(l) mfcolor(white) || /*

*/ rcapsym low_u low_l plot2, color(gs13) lcolor(gs13) s(i) lwidth(thin) || /*

*/ scatter low_p plot2, color(gs13) lcolor(gs13) s(t) c(l) mfcolor(white) || /*

*/ , graphregion(color(white)) ytitle(Percentage of all tests (95% CI) [log scale]) ylabel(0.001 0.003 0.005 0.01 0.03 0.05 0.1 0.3 0.5 1.0 0.0005 "0", angle(0) labsize(small)) yscale(log) /*

*/ xlabel(`min'(2)`max', valuelabels labsize(vsmall) angle(45)) xtitle("") /*

*/ legend(order(2 "Higher evidence" 4 "Moderate evidence" 6 "Lower evidence" ) size(vsmall) rows(1))

graph export "$Graphs\positives_over_wofy_fig4b.emf", replace

export excel wofy high_p high_l high_u mod_p mod_l mod_u low_p low_l low_u using "$Logs\Figure4B.xlsx", replace firstrow(var)

noi list wofy n *_p*, noobs nod

log close
